# Microenvironmental response-based treatment of osteoarthritis is a highly effective and durable program: a review

**DOI:** 10.1186/s13018-025-06399-3

**Published:** 2025-11-05

**Authors:** Yongbiao Meng, Shenghua Wu, Shile Liu, Yuefen Yang

**Affiliations:** Department of Orthopedics, First People’s Hospital of Linping District, Hangzhou, 311199 China

**Keywords:** OA, Disease pathogenesis, Microenvironmental response, Bio-materials

## Abstract

Osteoarthritis ( Agostini et al. in J Pers Med 14(8), 2024) is a major health concern causing disability in middle-aged and elderly populations, primarily characterized by degeneration and damage of articular cartilage. The pathological features of OA often overlap with normal aging processes, complicating clinical diagnosis. Studies have shown that OA progression is accompanied by dynamic changes in the internal microenvironment, making microenvironment-responsive diagnostic and therapeutic strategies a focus of research. This review provides a comprehensive overview of current pathological characteristics and microenvironmental factors associated with OA, including histopathological features, inflammatory microenvironment, extracellular matrix microenvironment, and epigenetic microenvironment, which involve complex multi-level interactions among cells, molecules, and signaling pathways. Furthermore, we systematically summarize current therapeutic approaches encompassing non-pharmacological therapies, pharmacological agents, herbal medicine, and surgical interventions, with particular emphasis on novel microenvironment-responsive therapeutic strategies, especially the cross-disciplinary applications of biomaterials in this field. This review aims to advance research on OA diagnosis, internal microenvironmental dynamics, and material-based microenvironment modulation, while offering new research perspectives for professionals in healthcare, biomaterials science, and clinical practice.

**Graphical abstract:**

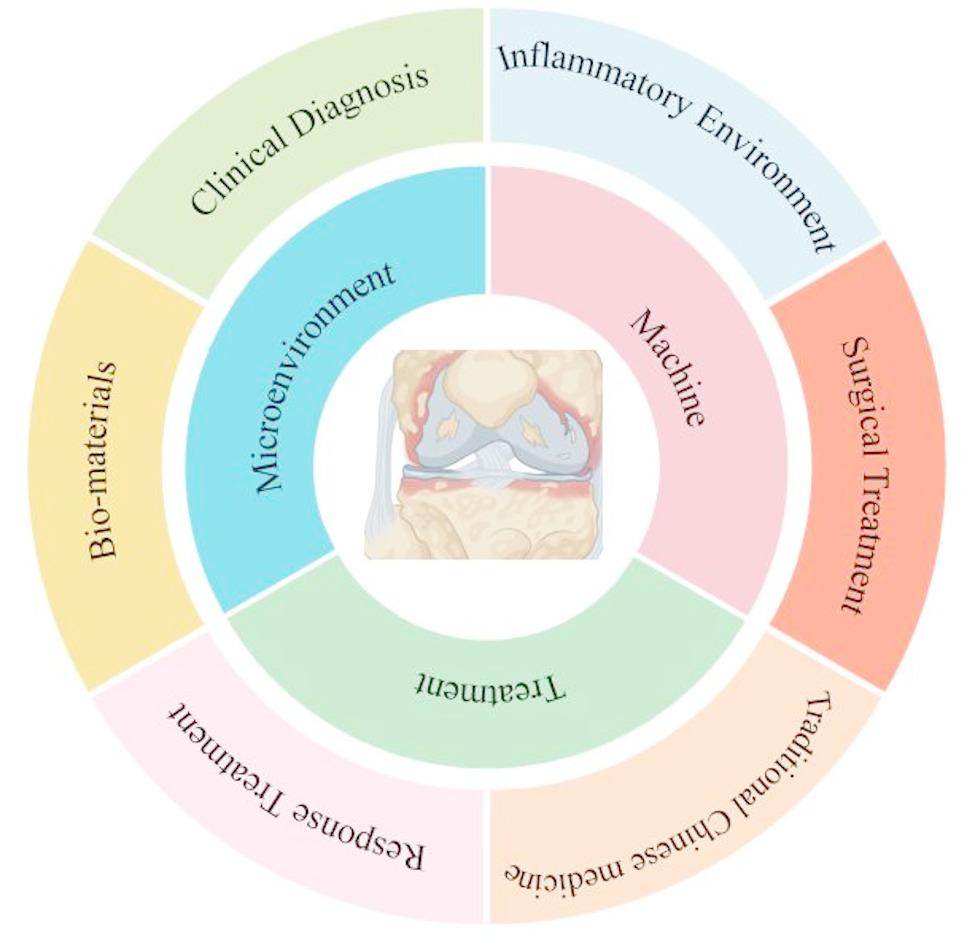

## Introduction

Osteoarthritis [1] is a prevalent chronic and disabling degenerative disorder characterized by structural joint deterioration and functional impairment, predominantly affecting weight-bearing joints such as the knee and hip. Its primary pathological manifestations include osteochondral degeneration, subchondral bone sclerosis, ligament laxity or contracture, and muscular atrophy and weakness [2]. Current epidemiological data indicate that osteoarthritis [1] affects approximately 10% of males and 18% of females aged over 60 years. In China, the OA patient population now exceeds 150 million individuals. OA is clinically associated with severe pain and progressive functional deterioration, resulting in activity and mobility limitations. These impairments not only impose significant physical and psychological burdens but also position OA as the second leading cause of global disability, underscoring its critical public health impact [3]. The pathogenesis of OA is mainly thought to be related to age, gender, genetics, occupation and lifestyle, etc [4]. Also pro-inflammatory factors in the body promote the production of protein hydrolases (e.g., cysteine proteases such as MMP, histone K, serine proteases) [5]. Furthermore, certain intrinsic biological mediators—including fibroblast growth factor (FGF) signaling transduction, bone morphogenetic protein (BMP) and Wnt signaling pathways, as well as epigenetic regulators—may exacerbate OA progression through dual mechanisms: activating pathways that promote articular tissue destruction or suppressing cellular repair of damaged extracellular matrices [6]. The limited regenerative capacity of articular cartilage constitutes a fundamental pathological driver of osteoarthritis [7] progression and serves as the primary etiological factor underlying end-stage joint functional impairment. Current therapeutic paradigms primarily focus on symptomatic pain relief, preservation or restoration of joint functionality, and structural protection, while implementing standardized yet personalized treatment strategies tailored to distinct clinical stages of OA development. These interventions encompass: Pharmacological management [8], Physical therapeutics [9], Intra-articular delivery systems [10], Surgical reconstruction [11].

Current intra-articular pharmacological interventions, while effective in symptom alleviation and pain management, fail to adequately promote osteochondral regeneration. Consequently, a subset of patients progresses to end-stage osteoarthritis [7], necessitating total joint arthroplasty for functional restoration [12]. Notably, although direct intra-articular drug delivery circumvents systemic toxicity, the unique synovial microenvironment—characterized by rapid synovial fluid turnover and enzymatic degradation—severely limits drug retention and bioavailability. This pharmacokinetic challenge impedes effective recruitment and proliferation of osteoprogenitor cells, ultimately compromising tissue repair outcomes [13]. Emerging insights into OA pathogenesis highlight the pivotal role of matrix metalloproteinases (MMPs) in disease progression [6]. Specifically, MMP-13 and MMP-3 mediate proteolytic degradation of critical extracellular matrix (ECM) components, including aggrecan, type II/IV/IX collagen, and osteonectin, thereby accelerating cartilage destruction [14]. Parallel studies implicate ADAMTS (a disintegrin and metalloproteinase with thrombospondin motifs) in aggrecanolysis, which destabilizes chondrocyte morphology and triggers apoptotic cascades, further exacerbating OA pathology [15]. Pharmacological inhibition of MMPs demonstrates therapeutic potential. For instance, oral administration of herbal extracts (e.g., curcuminoids and boswellic acids) downregulates MMP-3 and COX-2 gene expression, suppresses collagenase activity, and preserves articular cartilage integrity by maintaining collagen and proteoglycan content [16]. Notably, throughout the progression of osteoarthritis, the bone microenvironment undergoes consistent changes, including degenerative alterations in articular cartilage (such as softening, erosion, and exfoliation), secondary subchondral bone sclerosis, osteophyte formation, and synovial inflammation [1]. Recent advancements in biomaterial-based therapeutics offer innovative strategies to address OA’s dynamic microenvironment. Key pathological features include: Pro-inflammatory cytokine storms (e.g., IL-1β, TNF-α) driving synovitis [6], Proteolytic enzyme overexpression disrupting ECM homeostasis [15], Immune cell infiltration (e.g., M1 macrophages) perpetuating inflammation [17], Reactive oxygen species (ROS)-mediated chondrocyte apoptosis [18]. Notably, ROS-responsive nanoplatforms exemplify precision medicine approaches. A representative system employs poly(ethylene glycol)-b-poly(thioketal) (PTK) copolymers co-assembled with bortezomib (BTZ) to form drug-loaded nanoparticles. These nanoparticles passively target OA lesions via enhanced permeability and retention (EPR) effects, followed by ROS-triggered BTZ release. This strategy effectively repolarizes M1 macrophages to anti-inflammatory M2 phenotypes, reduces synovial hyperplasia, and attenuates cartilage degradation—demonstrating synergistic therapeutic efficacy [19].

Research on osteoarthritis is mainly focused on the pathogenesis, drug delivery and rehabilitation, which are independent and unrelated to each other. Although such research can alleviate the progression of OA in the short term, it cannot effectively promote bone regeneration and treat OA. In recent years, the development of microenvironmental mechanisms of OA pathogenesis and biomedical materials has led to a combination of orthopedic, biological, material, and mechanism, which has provided a new direction for the treatment of OA. This paper aims to expand these findings, improve the understanding of OA, and elucidate the interaction mechanisms, with a view to understanding the specific therapeutic mechanisms and potential directions for OA. Therefore, this article will summarize the research progress of OA in recent years. The properties of the OA microenvironment are analyzed. Finally, this review investigates some practical new applications of biomaterials as monomer drugs and nanocarriers in the field of drug delivery. This paper will provide some research help in the specific mechanism of OA, the specific mechanism of microenvironment improvement of metabolic diseases, and the improvement of drug utilization.

## OA

OA is a common chronic progressive degenerative joint disease that mainly affects articular cartilage, bones and surrounding tissues, severely affecting patients’ quality of life. The disease is characterized by joint pain, stiffness and limited movement as its main clinical features, and has become an important cause of dysfunction affecting the public. The complexity of its pathologic features and microenvironment is the main reason affecting its treatment.

### Pathological feature

OA is mainly accompanied by thickening of the subchondral bone, formation of bony encumbrances at the joint margins, and mild, chronic, nonspecific synovial inflammation [3]. Together, this series of pathologic changes leads to impaired joint function and pain. Loss of articular cartilage, which is the core problem in osteoarthritis, destroys the cushioning and lubrication of the joints, exposing them to greater friction and stress during movement [20]. At the same time, the thickening of the subchondral bone and the formation of bony encumbrances further affect the structure and stability of the joints [21]. Normal aging of the body is accompanied by this cartilage degeneration which is not clearly differentiated from the physiological changes in osteoarthritic cartilage, thus this makes the early diagnosis of osteoarthritis difficult. Currently, it is possible to distinguish normal cartilage, aged cartilage and osteoarthritic cartilage mainly by analyzing the different stages of cartilage [22]. For example, normal cartilage has a specific structure and function, aged cartilage is altered in some ways (such as collagen loss, structural disruption, and surface fibrillation), which has not yet reached the level of osteoarthritis, whereas osteoarthritic cartilage shows obvious pathological changes such as destruction of cartilage, formation of bone redundancy, and synovial inflammation [23].

Normal articular cartilage comprises chondrocytes embedded in an extracellular matrix (ECM) rich in type II/IX/XI collagens and aggrecan aggregates, conferring structural integrity and biomechanical resilience [24, 25]. Chondrocytes regulate ECM synthesis and turnover to maintain tissue homeostasis [26]. In osteoarthritis [7], pathological progression features triad degeneration: cartilage erosion, osteophyte formation, and subchondral sclerosis [27]. Histologically, OA evolves through three phases [28]. Initial stage: Mid-zone ECM edema with surface fibrillation, chondrocyte cluster formation, and focal cellular loss [29]. Intermediate stage: Collagen fiber disruption generating vertical fissures extending toward subchondral bone, accompanied by chondrocyte hypertrophy [30]. Advanced stage: Full-thickness cartilage denudation exposing sclerotic bone, with synovial inflammation triggered by joint debris [21, 31]. Fibrocartilage-covered osteophytes and trabecular bone remodeling further accelerate joint dysfunction [32].

OA pathogenesis represents a multifaceted interplay involving coordinated pathological alterations across articular cartilage, subchondral bone, and synovium. Elucidating these pathological hallmarks and their underlying molecular cascades provides critical insights for: (1) early-stage diagnostic biomarker discovery; (2) targeted therapeutic interventions; and (3) precision prevention strategies to mitigate OA progression, thereby optimizing clinical outcomes in OA management through mechanistically informed approaches.

### Microenvironment

**Fig. 1:**
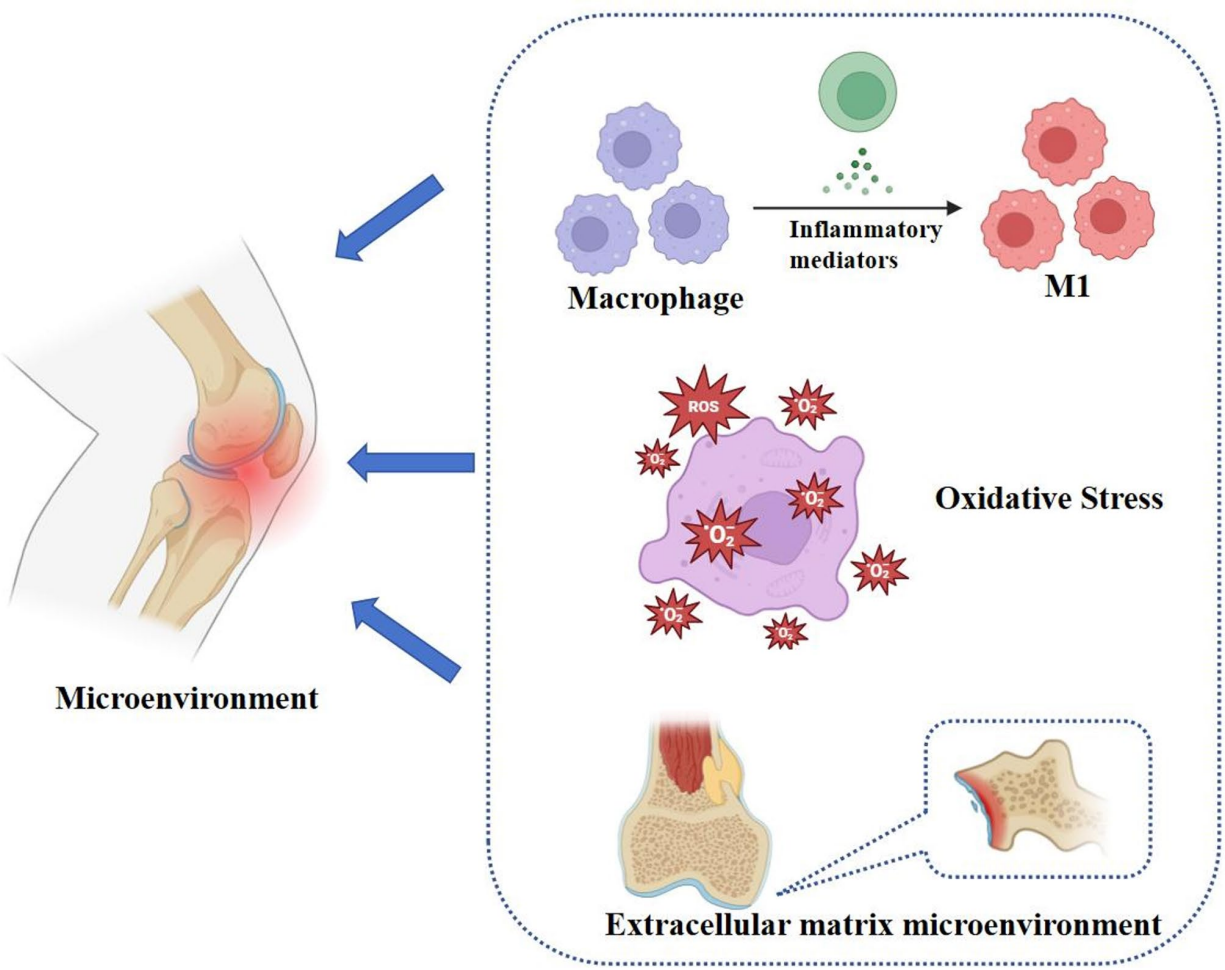
Osteoarthritis Microenvironment. The development of OA is accompanied by microenvironmental changes such as inflammation, oxidative stress, and extracellular matrix

The onset and progression of OA is accompanied by this very complex system of microenvironmental dynamics. It is involved in the interaction of multiple cellular, molecular and signaling pathways. An in-depth understanding of its mechanisms is important for the development of novel therapeutic strategies. Figure 1 shows the reasons for OA to occurred.

#### Inflammatory microenvironment

OA is accompanied with continuous inflammation, and the inflammatory microenvironment plays a key role in the pathogenesis and progression of osteoarthritis. Currently, cells such as macrophages, neutrophils and lymphocytes are able to stimulate chondrocytes to produce matrix-degrading enzymes and accelerate cartilage destruction through the production of a large number of cytokines and chemokines, thus contributing to the onset and progression of osteoarthritis [21]. Among these are macrophages, which are typically important inflammatory cells that migrate from the bloodstream into damaged joint tissues in the early stages of osteoarthritis, forming infiltrating [33]. In addition, their accumulation and activated forms in the joints, especially M1-type macrophages, exacerbate inflammation and cartilage damage, and they are able to promote local inflammatory responses through the production of inflammatory factors (TNF-α, IL-1β, IL-6, etc.), which at the same time not only promote the recruitment of inflammatory cells, but also exacerbate the apoptosis of chondrocytes and the degradation of the matrix [34]. Furthermore macrophages in the osteoarticular environment promote the degradation of the cartilage matrix through the secretion of enzymes such as matrix metalloproteinases (MMPs), leading to further damage to the cartilage, affecting its structure and consequently the biomechanical properties of the joints [35]. It has also been found that macrophages can also modulate the local immune response by secreting anti-inflammatory factors (e.g., IL-10), and this dual role gives macrophages a complex role in the progression of osteoarthritis, as well as the possibility that macrophages may undergo metabolic reprogramming that changes their energy metabolism to adapt to the inflammatory state [36]. Meanwhile, in the inflammatory microenvironment, dendritic cells participate in initiating joint inflammation and bone destruction by serving as key precursors to osteoclasts [37]. Activated T cells (particularly the Th17 subset) promote inflammation and osteoclast genesis by secreting pro-inflammatory cytokines such as IL-17, while regulatory T cells (Tregs) may attempt to suppress inflammation and promote repair [38]. Furthermore, osteoclasts become abnormally activated in the early stages of OA, resorbing subchondral bone, promoting vascular invasion, and altering the joint microenvironment, thereby disrupting bone homeostasis. Conversely, osteoblasts undergo reactive proliferation in the late stages of OA, leading to abnormal hardening of subchondral bone (osteophyte formation) and changes in joint structure [39].

Excessive production of reactive oxygen species (ROS) and abnormal ATP energy metabolism associated with the oxidative phosphorylation pathway in mitochondria are also closely related to OA. Factors such as hypoxia, stress load and chronic inflammation can stimulate chondrocyte mitochondria to produce large amounts of ROS, causing oxidative stress leading to structural damage of nucleic acids, proteins and lipids, resulting in mitochondrial dysfunction and chondrocyte metabolism disorders and even apoptosis [40]. ROS accumulation can also lead to elevated expression of inflammatory factors, chemokines, and matrix metalloproteinases, promoting cartilage matrix degradation and disrupting cartilage homeostasis [41]. In turn, disruption of the metabolism of mitochondrial capacity in the bone environment contributes to OA. Increased chondrocyte glycolytic activity in osteoarthritis leads to increased lactate production and decreased cartilage matrix synthesis. Mitochondria produce ATP through aerobic respiration, which is the primary source of cellular energy, and normal chondrocytes depend on mitochondrial function for their metabolic activity and survival. In osteoarthritis, the mitochondrial function of chondrocytes may be impaired, leading to a decrease in ATP production, thus affecting cell survival and function [42]. On the other hand, changes in mitochondrial membrane permeability lead to the release of pro-apoptotic factors that trigger cell death, and in osteoarthritis, apoptosis of chondrocytes is increased, which may be related to mitochondrial dysfunction and oxidative stress [43]. Additionally, abnormally elevated cholesterol and oxidized low-density lipoprotein can induce joint inflammatory responses, activate proteolytic enzymes, and accelerate chondrocyte apoptosis. Concurrently, impaired cholesterol reverse transport pathways lead to abnormal lipid deposition within chondrocytes, subsequently causing cell hypertrophy, ossification, and cartilage degeneration. Simultaneously, dysregulated adipokine secretion and abnormal fatty acid oxidation further exacerbate inflammation and cartilage matrix degradation, disrupting joint homeostasis [44].

#### Extracellular matrix microenvironment

The defects in articular cartilage were the root cause of the difficulty in treating OA. Among them, the microenvironment of cartilage is also closely related to the progression of OA.The progression of OA is often accompanied by biochemical changes and altered biomechanical properties of the extracellular matrix of cartilage [45]. In osteoarthritis, the composition and structure of the ECM are altered, leading to degradation of the cartilage matrix. Overexpression of matrix metalloproteinases (MMPs) and other enzymes destroys collagen and proteoglycans, leading to degradation of cartilage [46]. Alterations in the extracellular matrix can promote infiltration and activation of inflammatory cells, and certain components of the ECM (e.g., collagen fragments) can act as “red flags” to activate the immune response, leading to increased synovial inflammation [47]. ECM regulates cell adhesion, migration and proliferation by binding to integrins on the cell surface [48]. Osteoarthritis leads to changes in joint loading and movement patterns, which in turn affects the mechanical properties of the ECM [49]. Changes in mechanical stress affect cell signaling and gene expression, further exacerbating cartilage degradation. As osteoarthritis progresses, the regenerative capacity of the ECM decreases, leading to diminished cartilage repair. This is associated with a decrease in growth factors and cytokines in the ECM, affecting chondrocyte proliferation and differentiation.

In addition, as osteoarthritis progresses, the modulus of elasticity of cartilage usually decreases, leading to a reduction in its ability to withstand loads, which makes cartilage more susceptible to damage during joint movement [50]. Whereas the water content of cartilage in osteoarthritis patients may increase or decrease, which also affects the lubricity and cushioning capacity of cartilage, changes in water affect the mechanical properties of cartilage, leading to a decrease in its ability to deform under load [51]. In the OA setting, the load distribution in the joint becomes uneven, resulting in excessive stress on certain areas. This uneven load distribution accelerates cartilage degradation and may lead to the formation of bone spurs (osteophytes). It also leads to changes in joint alignment, such as inversion or valgus of the knee, which further affects load distribution and joint biomechanical properties [52]. Additionally joint fluid viscosity may increase, affecting joint lubrication and smooth movement, and changes in viscosity can lead to increased joint friction, which can accelerate cartilage wear [53].

Overall, extracellular microenvironmental changes in osteoarthritis are a complex process involving multiple aspects of cartilage, sub-bone structures, joint fluids, and movement patterns. These changes not only affect joint function and stability, but may also accelerate disease progression. Understanding these biological changes is essential for developing effective therapeutic strategies and preventive measures.

#### Epigenetic regulation

OA pathogenesis involves a variety of genetic, environmental, and biomechanical factors [4]. In recent years, studies in epigenetics have provided new perspectives for understanding the onset and progression of osteoarthritis. Epigenetics is the study of the regulation of gene expression and involves mechanisms such as DNA methylation, histone modification, and non-coding RNAs [54]. In patients with osteoarthritis, certain genes associated with cartilage metabolism, inflammatory response, and apoptosis may exhibit aberrant methylation status. For example, the promoter regions of some key genes (e.g., IL-1β, MMPs, etc.) in chondrocytes may be methylated, which inhibits their expression and promotes inflammation and matrix degradation [55]. Also with age, DNA methylation patterns change, which may be associated with an increased risk of osteoarthritis. Studies have shown that methylation levels of genes associated with cartilage metabolism in the elderly population may affect their function [56]. In osteoarthritis, changes in histone modifications may lead to abnormal expression of genes associated with inflammation, cell proliferation, and apoptosis. For example, an increase in H3K27me3 may be associated with the upregulation of the expression of certain repressive genes [57]. Some other transcription factors (e.g., NF-κB) influence the expression of related genes by regulating histone modifications [54]. MicroRNAs (miRNAs) play an important role in osteoarthritis, such as miR-140 and miR-146a in chondrocytes that may influence the progression of osteoarthritis by regulating the expression of genes related to inflammation and matrix degradation [58]. Alternatively long non-coding RNAs (lncRNAs) may influence gene expression by interacting with transcription factors or histone modifications, thus participating in cartilage metabolism and inflammatory responses [59]. It is worth noting that epigenetics is closely linked to the presence of environmental factors [4]. Environmental factors such as lifestyle, diet, and exercise can influence the risk of osteoarthritis through epigenetic mechanisms. For example, obesity and high-sugar diets may affect genes associated with inflammation and metabolism by altering DNA methylation and histone modifications.

## Multimodal treatment strategies for OA

**Fig. 2:**
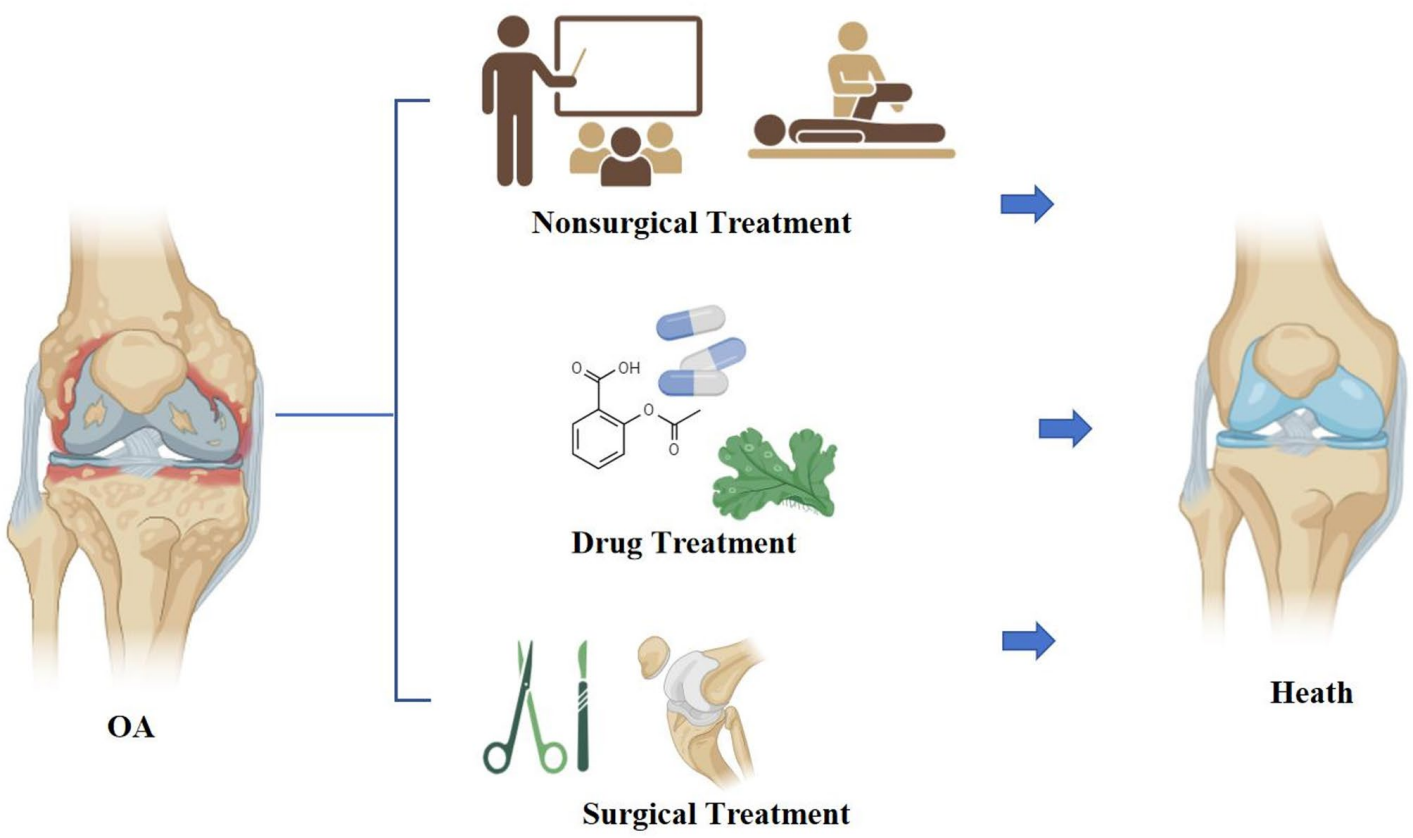
Osteoarthritis Treatment. The treatment of OA includes non-pharmacological approaches through educational outreach, medication using both traditional Chinese and Western medicine, as well as surgical intervention

OA seriously affects the health of the elderly population and has jumped to become the second most common disease-causing disability. As aging increases, how to effectively treat OA is attracting more and more attention. Currently, there are various treatment methods for osteoarthritis, covering a wide range of non-pharmacologic, pharmacologic and surgical means (Table 1; Fig. 2).

**Table 1:** Current treatment status of OA

Type of treatment	Model	Mechanism	Refs.
Basic treatment	Health education, exercise therapy, physical therapy and mobility support therapy.	Educate patients about managing osteoarthritis, including reasonable activity levels, proper posture, and avoiding unnecessary weight bearing. This helps patients understand how to reduce stress on the joints through daily activities.	[21]
		Physical therapy is a key part of osteoarthritis management and includes heat therapy, hydrotherapy, ultrasound, acupuncture, massage, traction and transcutaneous electrical nerve stimulation. These methods are designed to increase local circulation and reduce the inflammatory response and pain.	[60]
Drug treatment	Topical topical nonsteroidal anti-inflammatory drugs (NSAIDs), oral NSAIDs, articular cartilage protectors, intra-articular injections, anti-anxiety medications, and TCM medications	Direct action on the pain site, less systemic absorption, low gastrointestinal side effects. Suitable for mild to moderate pain. For example, diclofenac 150 mg/day is the most effective in terms of improving pain and function.	[61]
		Oral NSAIDs are currently the drug of choice for controlling OA-related symptoms and are recommended for OA patients with persistent pain symptoms or moderate-to-severe pain to reduce symptoms and improve function in people with OA. Celecoxib treatment resulted in decreased secretion of prostaglandins, Nesh-SH3 target (ABI3BP), and osteonectin, whereas secretion of tissue inhibitor of metalloproteinase 2 (TIMP-2) was significantly increased, and cartilage degeneration was significantly reduced.	[62]
		Chondroprotective agents possibly slow cartilage degradation and promote cartilage repair. Glucosamine sulfate treatment delayed collagen degeneration in cartilaginous discs, decreased serum TNF-α levels, and had a delaying effect on the progression of OA.	[63]
		Intra-articular injections provide short-term (2–4 weeks) pain relief, avoid accelerated cartilage degradation, and improve joint lubrication for early to mid-stage OA, while promoting cartilage repair and improving symptoms through anti-inflammatory and pro-repair effects. Intra-articular injections of hyaluronic acid significantly reduced spontaneous and evoked pain and improved disability. No serious systemic adverse events were reported. At up to 12-month follow-up, patients with OA treated with intra-articular hyaluronic acid injections reported improved pain relief and quality of life.	[1]
		TCM treatment for osteoarthritis can achieve individualized treatment through multi-targeted intervention (anti-inflammatory, antioxidant, and pro-repair), holistic conditioning, and fewer side effects, and can be combined with evidence-based treatment. Icariin can induce autophagy by regulating the PI3K/AKT/mTOR/ULK1 signaling pathway for the treatment of OA.	[64]
Microenvironmental Response Therapy	The development and progression of OA is accompanied by dynamic changes in the microenvironment of this very interaction involving multiple cellular, molecular, and signaling pathways,	The presence of an inflammatory microenvironment and degradation of the extracellular matrix produced by chondrocytes leads to increased cartilage damage and hinders osteoarthritis treatment. Chondroitin methacryloyl sulfate (ChsMA) was used as the core to design responsive inflammatory microenvironment microspheres. Reduces inflammation while promoting chondrocyte anabolism, favoring cartilage repair.	[65]
		High levels of reactive oxygen species (ROS) cause oxidative stress, leading to bone cell damage and death. A bifunctional hydrogel was designed to help inhibit inflammation by scavenging reactive oxygen species and provide lubrication to minimize joint wear.	[66]
		The pericellular matrix (PCM) plays a key role in signaling and mechanoprotection of chondrocytes, and specific matrix metalloproteinases are involved in PCM production and degradation. By matrix metalloproteinase 13 (MMP-13) response to a drug-carrying system, it was found to significantly attenuate oxidative stress, down-regulate the expression of hypoxia-inducible factor 1α and inflammatory cytokines, and prevent cartilage destruction.	[67]
Biomaterials	Biomaterials in OA treatment possess high biocompatibility, controlled degradability, and the promotion of cartilage regeneration and joint function restoration through drug slow release, tissue-engineered scaffolds, or nanotechnology.	Hydrogel with bionic repair and multifunctional integration for cartilage defect filling and inflammation modulation. Enhanced mechanical strength and remarkable injectability of hydrogels prepared using collagen while significantly promoting cell adhesion, proliferation and chondrogenic differentiation.	[68]
		Microspheres enable long-lasting drug delivery and cellular microenvironment support, making them suitable for long-term management of chronic OA. The hydration layer is introduced into the microspheres through a liposome-coated surface, and the microspheres form a self-renewable hydration layer through frictional wear to improve joint lubrication and maintain cellular homeostasis through increased autophagy.	[69]
		Microneedle is characterized by minimally invasive penetration and multimodal treatment, and is particularly suitable for precise intervention in deep joint pathologies. The prepared bilayer microneedles are capable of sustained, drug release through hydrolysis of the ester bond, physical diffusion of the tip, and breakthrough of the lubricating coating. At the same time, skin damage is reduced due to the presence of a lubricating coating on the surface.	[70]
Surgical treatment	OA surgical treatment corrects joint deformity, relieves pain quickly and restores joint function, with the advantages of less trauma, faster recovery and fewer complications.	Total Hip Arthroplasty Leads to Clinically Important and Better Hip Pain Reduction and Hip Function Improvement Compared to Resistance Training at 6 Months in Patients 50 Years of Age or Older with Severe Hip Osteoarthritis and Indication for Surgery.	[71]

### Non-pharmacological treatment

Current non-pharmacological management of OA primarily encompasses physical interventions, weight control, and assistive devices [21]. Patient education focuses on understanding OA pathophysiology, etiology, and self-management strategies, including avoidance of joint-stressing postures such as prolonged standing, kneeling, or squatting, as well as stair climbing reduction. Maintaining an optimal body weight is crucial for joint preservation and symptom alleviation [72], with evidence supporting calorie-restricted diets combined with exercise regimens for weight reduction [4]. Low-impact aerobic exercises (e.g., walking, cycling, tai chi) and muscle-strengthening protocols (quadriceps isometric contractions, straight-leg raises) enhance musculoskeletal protection and functional improvement [9, 60]. Appropriate utilization of mobility aids (canes, crutches, walkers) and joint orthoses demonstrates efficacy in enhancing local blood circulation, reducing inflammatory responses, and mitigating arthralgia.

### Medication

Currently, the main purpose of OA treatment drugs is to relieve symptoms, improve joint function and delay disease progression. The drugs used in the treatment of OA are mainly classified as analgesics, protective inhibitors, and proprietary Chinese medicines.

#### Chemical medicine

Contemporary pharmacological management of osteoarthritis [7] involves five principal categories: NSAIDs, injectables, chronic anti-inflammatory agents, disease-modifying antirheumatic drugs (DMARDs), and biological pathway inhibitors [73]. NSAIDs, including topical formulations (loxoprofen, flurbiprofen axetil, ketoprofen gels) and oral agents (ibuprofen, naproxen, diclofenac, celecoxib), provide anti-inflammatory, analgesic, and antipyretic effects [74]. Topical NSAIDs demonstrate comparable efficacy to oral formulations for acute musculoskeletal and mild-to-moderate OA pain, with reduced systemic adverse events, though limited in severe/chronic cases. Network meta-analysis identifies diclofenac 150 mg/day as the most effective and safest oral NSAID, though interindividual safety variations require further validation [61]. Injectable therapies (glucocorticoids, medical chitin derivatives, sodium hyaluronate) offer short-term pain relief and functional improvement but necessitate repeated administration with infection risks [75]. Oral chronic anti-inflammatory agents like glucosamine (essential for proteoglycan synthesis) and diacerein (reduces synovitis, enhances cartilage metabolism) exhibit sustained post-treatment effects and potential cardiovascular benefits, though contraindicated in chronic enteritis [76, 77]. DMARDs include methotrexate (prevents joint erosion, improves functionality in severe OA) and iguratimod (anti-inflammatory/immunomodulatory effects), the latter requiring hepatic monitoring [78, 79]. Biological inhibitors target pathways like PAR2 (AZ3451 attenuates chondrocyte apoptosis via autophagy modulation and P38/MAPK/NF-κB regulation) and FoxO transcription factors (panobinostat upregulates PRG4 while suppressing IL-1β-mediated inflammation) [80, 81].

#### Chinese medicines

Phytotherapeutic interventions for osteoarthritis [7] have demonstrated multi-target efficacy through both historical applications and modern pharmacological validation [82]. Botanical medicines like *Psoralea corylifolia*, *Drynaria fortunei*, and *Achyranthes bidentata* offer cost-effective alternatives to conventional therapies with enhanced therapeutic indices, attributed to their complex phytochemical profiles. Mechanistically, these agents modulate critical pathways including NF-κB, PI3K-Akt, and AMPK signaling. For instance, *Eucommia ulmoides* contains 73 bioactive constituents (flavonoids, iridoids, lignans) targeting 38 OA-related genes [83]. Recent advances in the gut-joint axis paradigm reveal novel therapeutic dimensions. Guizhi-Shaoyao-Zhimu decoction (GSZD) modulates intestinal microbiota composition, elevates beneficial bacteria (e.g., *Lactobacillus*), and reduces serum inflammatory markers (LPS, TNF-α, IL-6) while regulating 27 metabolic pathways involving fatty acids and glycerophospholipids [84]. Synergistic regimens combining herbal therapy with acupuncture or hyaluronic acid injections show enhanced clinical outcomes [85] Modern phytochemistry identifies bioactive compounds (polysaccharides, polyphenols, alkaloids) as key mediators of OA protection. Pueraria lobata polysaccharide (PPL-1) demonstrates dose-dependent chondroprotection (1.25–10 µg/mL) by suppressing TNF-α/IL-1β/IL-6 and inhibiting chondrocyte apoptosis in vivo [86]. Genistein from soybeans attenuates IL-1β-induced catabolism via NOS2/COX-2/MMP suppression while activating Nrf-2/HO-1 antioxidant pathways in human chondrocytes [87]. Notably, while botanical therapies exhibit multi-modal anti-inflammatory and metabolic regulatory properties, their clinical translation requires rigorous standardization to address phytochemical complexity and batch-to-batch variability. Future research should prioritize compound purification, pathway-specific efficacy validation, and pharmacokinetic optimization.

### Surgical treatment

Current surgical management of osteoarthritis [7] comprises three principal modalities. (1) Arthroscopic Debridement. This minimally invasive technique enables intra-articular visualization and removal of inflammatory mediators (cartilage debris, synovial microcrystals, catabolic cytokines) via subcentimeter incisions. It effectively disrupts inflammatory cascades while preserving periarticular tissues, with advantages including rapid recovery (ambulation within 24 h), low cardiopulmonary impact, and suitability for elderly/comorbid patients under local anesthesia. Primarily indicated for early-stage OA to alleviate mechanical symptoms and improve functional capacity [88]. (2) Joint Arthroplasty. The gold standard for end-stage OA involves prosthetic replacement of degenerated articular surfaces to restore biomechanical alignment and eliminate pain. Contemporary total knee arthroplasties demonstrate 15–20 year survivorship in >60% cases, though complications include periprosthetic fracture (3.2%), aseptic loosening (1.8%/year), and thromboembolism (1.3%). Postoperative rehabilitation typically achieves 90° flexion by week 2, with 85% patients reporting functional improvement exceeding preoperative expectations [89]. (3) Osteotomy. This joint-preserving strategy corrects axial malalignment (varus/valgus deformities) through controlled bone resection, redistributing mechanical loading from damaged compartments. While permitting high-impact activities post-recovery, 18–25% patients report residual medial joint-line tenderness. Optimal outcomes require precise patient selection based on age (< 55 years), BMI < 30, and localized cartilage wear (Kellgren-Lawrence grade ≤ III) [90]. Surgical decision-making necessitates comprehensive evaluation of biomechanical parameters (limb alignment, joint congruence), metabolic factors (BMI, bone quality), and functional demands.

## Comprehensive treatment

### Microenvironmental

The development and progression of OA is accompanied by dynamic changes in the microenvironment of this very interaction involving multiple cellular, molecular, and signaling pathways, and therefore microenvironmental response-based therapy is more effective. Inflammatory microenvironment presence and degradation of extracellular matrix produced by chondrocytes leads to increased cartilage damage and hinders treatment of osteoarthritis. As Miao et al. were inspired by the structure of chocolate peanuts, we developed an injectable environmentally responsive bilayer hydrogel microsphere using microfluidic technology. The microspheres were loaded with celecoxib (CLX) liposomes (ChsMA + CLX@Lipo@GelMA) using methacryloyl chondroitin sulfate (ChsMA) as the core and methacryloyl gelatin (GelMA) as the shell. CLX was released from the liposomes as the GelMA shell rapidly degraded in response to the osteoarthritic microenvironment and inhibited the production of inflammatory factors, suggesting a beneficial effect of the shell in reducing inflammation. Degradation of the internal methacryloyl microsphere core is accompanied by the release of chondroitin sulfate, which promotes chondrocyte anabolism and facilitates cartilage repair [65]. In a parallel study, Li et al. developed a bionic nanosystem targeting the pathological microenvironment characterized by lubrication failure in articular cartilage and severe joint capsule inflammation. This innovative system demonstrates dual functionality: enhanced lubrication performance and stimuli-responsive drug release. Their research revealed that polymer-modified nanosheets exhibit exceptional near-infrared absorption capacity, maintaining long-term lubrication stability under various mechanical conditions with a 75% reduction in friction coefficient compared to H_2_O. The system achieves controlled drug loading with a high diclofenac sodium [60] payload of 29.2%, enabling near-infrared-regulated responsive and sustained drug release. Cellular experiments demonstrated effective internalization of the lubricating nanosystem via endocytosis. Anti-inflammatory evaluations confirmed its therapeutic efficacy in mitigating osteoarthritis progression through dual regulatory mechanisms: upregulation of cartilage anabolic genes (collagen II and aggrecan) coupled with downregulation of catabolic proteases (MMP-13 and ADAMTS-5) and pain-related genetic markers (NGF and TNF-α) [91]. Microenvironmental changes in metallo-matrix enzymes have also been shown to be critical in the diagnosis and treatment of OA. For example, Danalache et al. studied 148 cartilage samples from OA patients to explore the role of matrix metalloproteinases (MMP) in catabolism, and found that MMP-2, -3, and − 7 were recognized as important contributors to early bone degradation, disrupting key components such as collagen type VI and basement membrane proteins [46]. While Zhou et al. chose matrix metalloproteinase 13 (MMP-13) as the response site and prepared an injectable hydrogel microsphere system (HAM-SA@HCQ) modified by methacrylate in hypoxic inflamed joints, and found that the hydrogel microspheres exhibited strong drug-carrying capacity, prominent reactive oxygen species (ROS) scavenging ability, and specific hypoxia-responsive drug-releasing ability. In the OA tissue microenvironment, hydrogel microspheres were degraded by excess MMP-13 under hypoxic conditions and released HCQ, which synergized with ROS-scavenging cuproaromatics to inhibit the inflammatory response of macrophages. HAM-SA@HCQ injected into OA inflamed joints significantly attenuated oxidative stress, down-regulated the expression of hypoxia-inducible factor 1α and inflammatory cytokines, and prevented cartilage destruction [67].

The degradation of articular cartilage is mechanistically linked to the generation of matrix-derived bioactive fragments capable of integrin activation. Emerging evidence suggests that integrin signaling may orchestrate protease-mediated matrix remodeling through a feedforward mechanism in response to osteoarthritic matrix alterations. In this study, primary human chondrocytes stimulated with fibronectin (FN) fragments - known ligands for α5β1 integrin - demonstrated that clathrin-mediated endocytosis of integrin-FN fragment complexes drives MMP-13 (matrix metalloproteinase 13) production via redox-sensitive pathways. Notably, unlike intact FN molecules, FN fragments specifically activated α5β1 integrin through a spatiotemporally regulated process: initial plasma membrane-localized reactive oxygen species (ROS) generation followed by sustained ROS production within early endosomal compartments. These ROS-generating endosomes, termed “redoxosomes”, serve as signaling platforms containing three critical components: (1) internalized integrin-FN fragment complexes, (2) the enzymatic ROS source NADPH oxidase 2 (NOX_2_), and (3) SRC kinase, which functions as both a redox sensor and effector to promote MMP-13 transcription [92]. Emerging evidence reveals a redox-active endosomal signaling cascade mediating α5β1 integrin signal transduction and its pathological effector role in promoting matrix metalloproteinase (MMP) production within osteoarthritic chondrocytes. This mechanistic understanding highlights the therapeutic relevance of targeting microenvironment-responsive epigenetic alterations. Yang et al. elucidated a biomechanical-epigenetic regulatory axis wherein pathological mechanical stress induces extracellular matrix (ECM) disruption and chondrocyte phenotypic shift, driving anabolic-catabolic homeostasis imbalance favoring catabolic activation. Notably, the emerging paradigm identifies microRNAs (miRNAs) as critical mechano-responsive regulators in OA pathogenesis. Their seminal work demonstrated that mechanical overloading upregulates miR-365 in growth plate chondrocytes, functioning as a mechanotransduction effector to promote hypertrophic differentiation. This epigenetic modulation exhibits dual pathological consequences: (1) transcriptional activation of matrix-degrading enzymes including MMP13 (matrix metalloproteinase 13) and collagen X (Col X), and (2) establishment of disease-specific molecular signatures characterized by miR-365 overexpression in OA cartilage and its hyperresponsiveness to inflammatory mediators like IL-1β. Importantly, miR-365 inhibition effectively attenuates IL-1β-induced MMP13/Col X expression, positioning targeted modulation of chondrocytic miR-365 through antisense oligonucleotides as a promising epigenetic intervention strategy for OA prevention and disease-modifying therapy [93]. The persistent low-grade intra-articular inflammation represents a well-established pathological driver that disrupts synovial joint homeostasis, precipitating anabolic-catabolic imbalance in articular cartilage. Emerging evidence positions epigenetic microRNAs (miRNAs) as critical regulators of inflammatory signaling cascades, with dysregulated miRNA expression profiles establishing a mechanistic link to OA pathophysiology. Systematic characterization of IL-1β-induced transcriptional reprogramming in chondrocytes identified miR-146a-5p as the most responsive miRNA to pro-inflammatory stimulation. Functional validation revealed that genetic ablation of miR-146a-5p significantly attenuated IL-1β-mediated inflammatory responses and catabolic processes in vitro, while chondrocyte-specific miR-146a silencing ameliorated cartilage degradation and alleviated OA-associated pain phenotypes in surgically-induced murine OA models. Complementary RNA-seq profiling demonstrated IL-1β-responsive differentially expressed genes (DEGs) enriched in pathways governing inflammatory responses (NF-κB signaling), extracellular matrix organization (aggrecan metabolism), and cellular energetics (mitochondrial oxidative phosphorylation). Through an integrated systems biology framework combining miRNA target prediction (TargetScan, miRDB) and protein interaction network analysis (STRING database), miR-146a-5p was computationally mapped to regulatory nodes coordinating inflammation resolution (TRAF6/IRAK1 axis) and cartilage homeostasis maintenance (SOX9-COL2A1 pathway) [94].

### Biomaterials

**Fig. 3:**
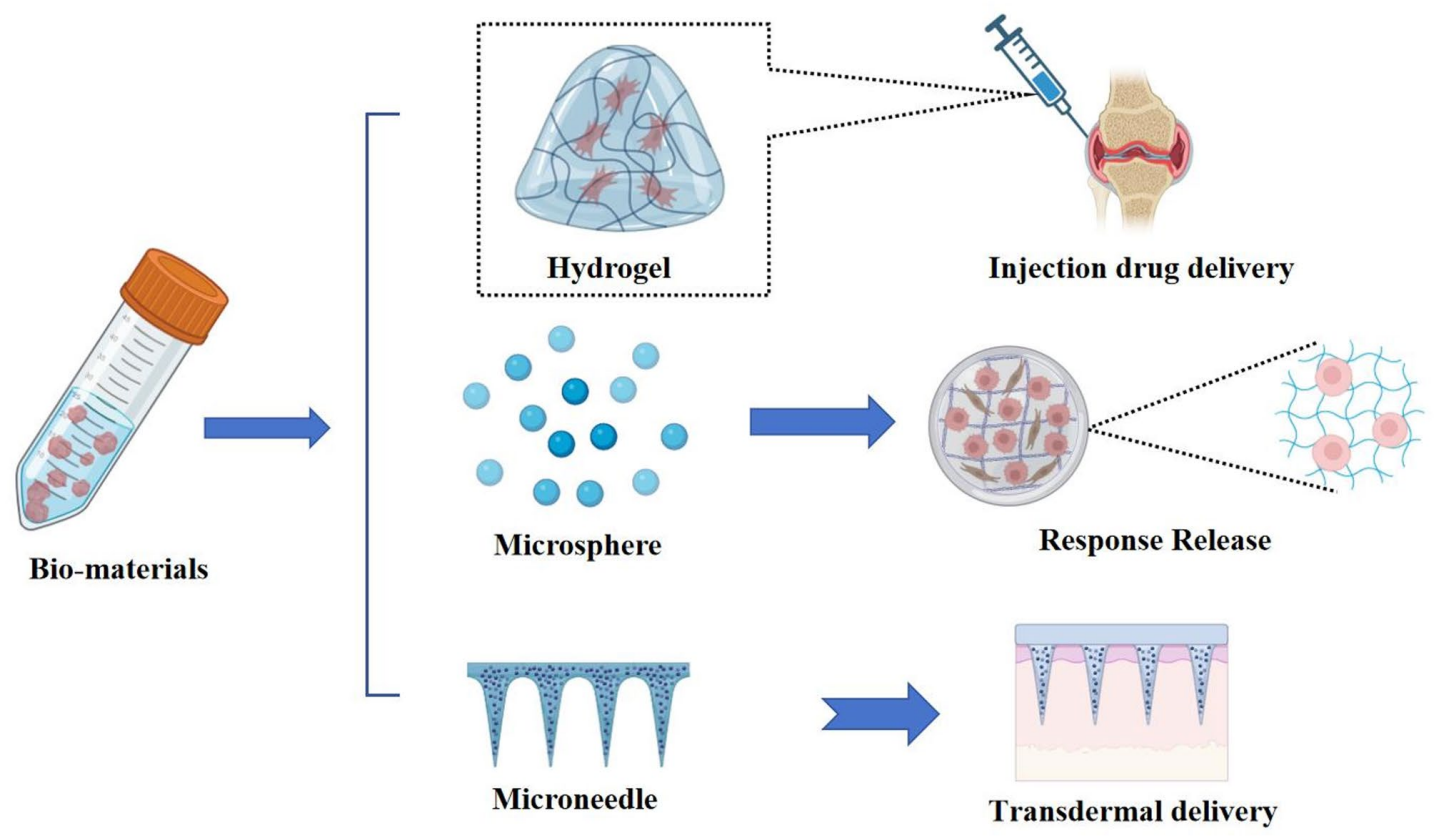
Biomaterials for Osteoarthritis. Hydrogels, microspheres, and microneedles prepared from biomaterials have been used in the treatment of OA

With the advancement of biomaterials and tissue engineering, polymeric materials have been extensively applied in bone regeneration and therapeutic interventions. Approaches such as hydrogels, microspheres, and microneedles are widely utilized in bone-related applications. Additionally, the modifiability and excellent biocompatibility of polymeric materials offer potential for personalized customization in bone treatments, enabling tailored solutions for diverse clinical needs (Fig. 3).

Currently, hydrogels are primarily applied in osteoarthritis [7] treatment through injectable hydrogels and stimuli-responsive hydrogels. These hydrogels enable efficient therapeutic delivery to transform OA treatment approaches. For instance, Lei et al. developed a bifunctional injectable hydrogel for OA therapy. The hydrogel was fabricated by modifying hyaluronic acid with 3-aminophenylboronic acid (PBA), followed by crosslinking it with hydroxyl-containing polyvinyl alcohol (PVA) to construct a dual-dynamic covalent crosslinked hydrogel (oHA-PBA-PVA gel). Experimental results demonstrated that this hydrogel effectively scavenges reactive oxygen species (ROS) to suppress inflammation and provides lubrication to reduce joint wear [66]. In the development of polymeric hydrogel materials, innovative approaches such as protein-based hydrogels and superlubricating hydrogels have also been explored. These hydrogels exhibit potential for reducing joint friction and inflammation. For example, Chen et al. designed a novel hydrogel system using collagen, which was crosslinked via thiol-mediated chemistry with cerium oxide (CeO_2_) and iron oxide (Fe_2_O_3_) nanoparticles encapsulated in poly(D, L-lactide-co-glycolide) (PLGA) microspheres (termed CSH-CeO_2_–pFe_2_O_3_). This system was engineered for controlled release of CeO_2_ and Fe_2_O_3_ nanoparticles. It demonstrated enhanced mechanical strength, remarkable injectability, and significantly promoted cell adhesion, proliferation, and chondrogenic differentiation [68]. Hou et al. developed a zwitterionic lubricating hydrogel for osteoarthritis [7] treatment. This hydrogel was designed and fabricated via microfluidic technology, utilizing free radical polymerization of sulfobetaine [95]-modified hyaluronic acid methacrylate. The resulting copolymer contained abundant SB and carboxyl groups, which provided superlubrication through hydration and formed electrostatic loading interactions with metformin (denoted as Met@SBHA), achieving high drug-loading capacity to counteract chondrocyte senescence [96]. Furthermore, in the development of hydrogels, recent studies have demonstrated that integrating bioactive compounds into hydrogel matrices can significantly enhance therapeutic efficacy. For instance, Zhang et al. engineered a dual-responsive hydrogel sensitive to pH and reactive oxygen species (ROS) by encapsulating quercetin (Que) within zeolitic imidazolate framework-8 (ZIF-8). This system effectively balanced lubrication and controlled release of bioactive agents, thereby modulating the bone tissue microenvironment to alleviate synovitis and cartilage matrix degradation while promoting cartilage regeneration [97]. Similarly, Yang et al. incorporated the bioactive compound baicalin (Bai) into a hydrogel system. This design leverages the selective uptake of Bai by fibroblast-like synoviocytes (FLS) to modulate glycolytic activity, concurrently suppressing the secretion of pro-inflammatory cytokines, including interleukin-1β (IL-1β), IL-6, and IL-8. Furthermore, the functional hydrogel (denoted as AG-Pm-OC) served dual roles as a lubricant and a nutrient-deprivation agent, effectively prolonging intra-articular retention of Bai. This synergistic strategy significantly attenuated cartilage degradation and enhanced synovial inflammation suppression [98]. Hydrogel-based systems for OA treatment have emerged as a focal point of contemporary research. Advancing understanding of hydrogel materials has unlocked diverse therapeutic avenues to enhance OA treatment efficacy, particularly through the rational design of their physicochemical properties and multifunctional integration. This field continues to evolve, offering innovative solutions to address pathological mechanisms.

Microsphere-based platforms are pivotal in osteoarthritis [7] therapy for cellular support, recapitulating native tissue microenvironments in vitro and in vivo while serving as efficient delivery systems for sustained release of therapeutics or biologics, ultimately enhancing cellular proliferation, migration, and differentiation [99]. Current microsphere fabrication primarily employs polymers (e.g., polylactic acid, polyvinyl alcohol), inorganic materials (e.g., silica, hydroxyapatite), and biomaterials (e.g., collagen, gelatin) through physical, chemical, or biological methods. These microspheres exhibit tailored biocompatibility, controlled release kinetics, and high drug-loading capacity. Precise modulation of size, morphology, and surface properties via material composition and processing enables OA-specific applications. For instance, Han et al. immobilized Nanofat [14] in aldehyde-modified PLGA porous microspheres (PMs) using Schiff base condensation and non-covalent interactions within a 3D porous network (PMs@NF). This design enhanced NF retention in cartilage (80% friction reduction), improved lubrication, and activated NF-derived stem cells via 3D penetration structures to upregulate anabolic genes while suppressing catabolic, inflammatory, and pain-related pathways [100]. Lipid-coated hydrogel microspheres (HMs) can reduce friction in OA via surface hydration layers, yet suffer lubrication failure upon coating damage. Lei et al. engineered rapamycin-loaded lipid-hyaluronate HMs (RAPA@Lipo@HMs) through microfluidic photopolymerization. This system employs a smooth rolling mechanism where friction-induced lipid exposure continuously regenerates hydration layers, achieving self-renewable lubrication while maintaining therapeutic payload integrity [69]. Microsphere-based systems demonstrate superior therapeutic profiles over conventional drug regimens, achieving comparable or enhanced efficacy within equivalent treatment durations while enabling reduced dosing frequency. This paradigm shift significantly alleviates therapeutic burden, thereby improving patient adherence through minimized procedural discomfort.

Microneedle [101] technology opens up new avenues for transdermal delivery of arthritis medications due to its painless skin perforation and efficient topical delivery compared to traditional oral or injectable delivery, which can cause gastrointestinal side effects [102]. For example, Chen et al. prepared a bilayer soluble MNs system by specifying a composite of hyaluronic acid (HA) and the covalently coupled drug compound diclofenac (DCF) as the tip of the MNs and then modifying the surface of the MNs tip with nanoparticles, and found that the system mainly achieves sustained drug DCF through the hydrolysis of the ester bond, the physical diffusion of the MNs tip, and the breakthrough of lubricating coatings release, while reducing skin damage and down-regulating the expression level of pro-inflammatory factors to alleviate articular cartilage damage destruction [70]. Microneedle systems can achieve the ability to achieve different drug release profiles through different self-enhancement methods or doping of nanocarriers. For example, in response to the poor aqueous solubility of Tet, which leads to low oral bioavailability and inconvenient injectable drug delivery, nanoparticles with immunoconformity and acid-responsive properties were formed by hybridization of poly(ethylene glycol)-denatured star-shaped PLGA with calcium carbonate, which increased the loading amount of Tet, and the nanoparticles were integrated into soluble microneedles prepared from peach gum, which had higher mechanical hardness and better physical stability, and this microneedle delivery strategy significantly Increased synovial uptake of Tet, which was further enhanced by co-functionalization of phagocytic stealth escape and inflammatory acidity-triggered release, while preventing local inflammation [103]. Currently MN shows clear advantages in delivering biological drugs or drugs that require local or frequent injections, filling a gap in the traditional delivery methods for arthritis drugs.

In summary, advancements in biomaterials have diversified therapeutic approaches for osteoarthritis [7], particularly in transdermal drug delivery, sustained release systems, nanoscale delivery platforms, and stimuli-responsive release technologies. However, most studies on biomaterial-mediated OA drug delivery have predominantly remained at the preclinical stage, with limited clinical translation. Future research must prioritize clinical trials to validate the efficacy, biosafety, and long-term biocompatibility of these materials. Furthermore, systematic investigations into the biomechanical properties required for intra-articular deployment remain scarce. Effective drug delivery to the joint cavity necessitates biomaterials with optimized mechanical resilience and thickness profiles to overcome cutaneous and intra-articular barriers while maintaining structural integrity during administration.

## Challenges and summary

Osteoarthritis [7] is a heterogeneous disorder influenced by multiple factors including age, body weight, genetic predisposition, and diverse molecular mechanisms. The increasing prevalence of this syndrome in recent decades has been closely associated with global population aging and the obesity pandemic. This review systematically summarizes the pathological characteristics and microenvironmental alterations in OA progression, evaluates current pharmacological interventions (both Western and traditional Chinese medicines), and particularly highlights emerging microenvironment-responsive therapeutic strategies, with a focus on interdisciplinary applications of biomaterials. Nevertheless, critical challenges persist in OA diagnosis and treatment that warrant further investigation.

Early OA detection remains problematic as conventional imaging modalities (X-ray and MRI) lack sensitivity in identifying initial articular structural changes, particularly subtle cartilage lesions and extracellular matrix compositional alterations. Current clinical biomarkers demonstrate insufficient specificity, while circulating biomarkers exhibit limited diagnostic accuracy. The complex pathogenesis involving mechanical stress, metabolic dysregulation in obesity, and miRNA-mediated genetic abnormalities further complicates early diagnosis. Current diagnostic reliance on late-stage symptoms (e.g., pain and limited mobility) often delays therapeutic intervention. Future diagnostic advancements should integrate high-precision imaging analyses with large-scale omics databases to identify OA-specific biomarkers for early preventive strategies. The OA joint microenvironment features chronic inflammation driven by multifaceted pathological processes: cartilage degeneration, synovitis, subchondral bone sclerosis, metabolic abnormalities, and oxidative stress. Pro-inflammatory cytokines (e.g., IL-1β and TNF-α) accelerate extracellular matrix degradation through upregulation of metalloproteinases, coupled with progressive loss of collagen and proteoglycans that impair cartilage self-repair. Cellular heterogeneity, manifested as region-specific chondrocyte phenotypes and polarized macrophage subpopulations, significantly influences therapeutic outcomes. Emerging microenvironment-targeted approaches aim to modulate macrophage polarization towards M2 phenotype, suppress pro-inflammatory gene expression, and enhance mesenchymal stem cell-mediated chondrogenic differentiation. Smart drug delivery systems responsive to pH, reactive oxygen species (ROS), and enzyme concentrations represent promising strategies for dynamic therapeutic adjustment.

Current pharmacotherapy primarily relies on NSAIDs and cytokine blockers for symptomatic relief. While effective in short-term pain and inflammation control, long-term administration may exacerbate gastrointestinal complications and cartilage deterioration. Next-generation therapeutics should concurrently address analgesia, anti-inflammation, and cartilage regeneration, with particular attention to novel molecular targets and bioactive compounds from traditional medicines. Surgical interventions require optimization regarding trauma minimization and postoperative cartilage rehabilitation. Biomaterial-based approaches offer novel solutions for targeted drug delivery, stimuli-responsive therapy, and sustained treatment. However, challenges persist including mechanical mismatch, suboptimal biodegradation-regeneration coordination, biocompatibility concerns from foreign body reactions, and translational barriers related to animal model limitations and high production costs. Future biomaterial development must prioritize biosafety evaluation, multifunctional design, and standardization of manufacturing protocols to accelerate clinical translation.

Interdisciplinary integration of dynamic microenvironment modulation with multimodal technologies holds great potential to overcome current OA management limitations. Advancements in early diagnosis, precision therapeutics, and regenerative strategies may ultimately achieve effective OA intervention and functional joint reconstruction.

## Data Availability

The datasets used to support the findings of this study are available from the corresponding author upon request.
